# Molecular and Phenotypic Characterization of a Highly Evolved Type 2 Vaccine-Derived Poliovirus Isolated from Seawater in Brazil, 2014

**DOI:** 10.1371/journal.pone.0152251

**Published:** 2016-03-28

**Authors:** Klécia Marília S. de Melo Cassemiro, Fernanda M. Burlandy, Mikaela R. F. Barbosa, Qi Chen, Jaume Jorba, Elayse M. Hachich, Maria I. Z. Sato, Cara C. Burns, Edson E. da Silva

**Affiliations:** 1 Enterovirus Laboratory, Oswaldo Cruz Institute, Oswaldo Cruz Foundation, Rio de Janeiro, Rio de Janeiro, Brazil; 2 Environmental Analysis Department, Environmental Company of São Paulo State, São Paulo, São Paulo, Brazil; 3 Division of Viral Diseases, Centers for Disease Control and Prevention, Atlanta, Georgia, United States of Americaa; The Scripps Research Institute, UNITED STATES

## Abstract

A type 2 vaccine-derived poliovirus (VDPV), differing from the Sabin 2 strain at 8.6% (78/903) of VP1 nucleotide positions, was isolated from seawater collected from a seaport in São Paulo State, Brazil. The P1/capsid region is related to the Sabin 2 strain, but sequences within the 5'-untranslated region and downstream of the P1 region were derived from recombination with other members of Human Enterovirus Species C (HEV-C). The two known attenuating mutations had reverted to wild-type (A481G in the 5'-UTR and Ile143Thr in VP1). The VDPV isolate had lost the temperature sensitive phenotype and had accumulated amino acid substitutions in neutralizing antigenic (NAg) sites 3a and 3b. The date of the initiating OPV dose, estimated from the number of synonymous substitutions in the capsid region, was approximately 8.5 years before seawater sampling, a finding consistent with a long time of virus replication and possible transmission among several individuals. Although no closely related type 2 VDPVs were detected in Brazil or elsewhere, this VDPV was found in an area with a mobile population, where conditions may favor both viral infection and spread. Environmental surveillance serves as an important tool for sensitive and early detection of circulating poliovirus in the final stages of global polio eradication.

## Introduction

The oral poliovirus vaccine (OPV), developed by Albert Sabin, has been effectively used for years in the control of poliomyelitis and elimination of wild polioviruses (WPV). Through its extensive use in mass vaccination campaigns, as part of the World Health Organization's (WHO) Global Polio Eradication Initiative (GPEI), it was possible to reduce the annual global incidence of polio from hundreds of thousands of cases to less than 70 in 2015, and now the WPV circulation is restricted to only two countries, Afghanistan and Pakistan (http://www.polioeradication.org/Dataandmonitoring/Poliothisweek.aspx).

Although OPV presents many advantages (easy administration, low cost, effective intestinal immunity and durable humoral immunity), Sabin strains are inherently genetically unstable [1, 2]. Due to the plasticity and rapid evolution of poliovirus genomes during replication in human gut, the strains evolve by reversion of known attenuating mutations and recombination with other members of Human Enterovirus C Species (HEV-C), leading to phenotypic changes and an increase in neurovirulence [3–6]. As a consequence of the genetic instability of OPV strains, vaccine-derived polioviruses (VDPV) have emerged on occasion in immunodeficient patients or under conditions of low population immunity, low vaccine coverage, poor sanitation and tropical conditions [7–9].

VDPVs are vaccine-related isolates whose genetic divergence from the parental OPV strains indicates prolonged replication or circulation [10,11]. Gaps in vaccination coverage may allow for circulation and antigenic drift of OPV strains. Type 1 and type 3 isolates that are >1.0% divergent and type 2 isolates that are >0.6% divergent in VP1 sequences from the corresponding Sabin strain are classified as VDPVs [12]. Ultimately, VDPV are particularly important for GPEI strategies, since the divergent strains regained the ability to cause paralytic polio in humans and the potential for sustained circulation, similar to wild-type virus, with a direct impact on polio eradication [1,10].

VDPVs are categorized as: 1) Circulating VDPVs (cVDPV), related to person-to-person transmission, 2) Immunodeficiency-associated VDPVs (iPVDV), isolated from individuals with primary immunodeficiency, and 3) Ambiguous VDPVs (aVDPV), which are isolates that cannot be classified definitively because they have no known source [10,12]. In recent years, 24 cVDPV outbreaks were identified in 21 countries, resulting in more than 750 cases of paralytic poliomyelitis [13].

Brazil has a decades-long record of elimination of WPV transmission, and no report of wild-type paralytic poliomyelitis since 1989 [14]. In January 2014, a highly evolved type 2 VDPV was isolated from seawater, during environmental surveillance in São Sebastião Seaport, located on the north coast of São Paulo State, in the Southeast of the country. This seaport is Brazil’s biggest port for liquid bulks, handling 26% of all of Brazil’s liquid cargos and counting with a high flow of vessels and people from all over the world. No VDPV had been reported from the environment or clinical samples in the Brazilian territory before or after this event, and no paralytic cases were linked to this isolate. The origin of the virus and the shedding individual are unknown, and calculations indicate that the original Sabin dose was given more than 8 years ago. In this report, we describe the genome characterization, phenotypic features and phylogenetic analysis of this highly divergent virus, and emphasize the need for sustained environmental surveillance, even in countries with a long period of interruption of indigenous transmission of WPV.

## Material and Methods

### Virus Isolation

L20B (NIBSC Accession No. 081102), RD (NIBSC Accession No. 081003) and HEp-2 (NIBSC Accession No. 740502) cell lines were provided by the WHO Global Polio Laboratory Network (GPLN) and used for virus isolation. The Oral Poliovaccine Sabin Type 2 reference strain (NIBSC code 01/530) was also provided by WHO.

The Environmental Company of the São Paulo State (CETESB) is the state government agency responsible for diagnosis and monitoring of environmental quality at São Paulo State, Brazil. CETESB works in the field of Environmental Virology for about 40 years and since 1999 develops a Surveillance Program for Enterovirus in collaboration with the Center of Epidemiological Surveillance of São Paulo State. The surveillance is based on bi-weekly routine examination of sewage samples from potential points of foreign people such as international airport and seaports, and from wastewater treatment plants. All poliovirus isolated are referred to the Enterovirus Laboratory at Oswaldo Cruz Foundation in Rio de Janeiro, Brazil (WHO Regional Reference Laboratory) for further characterization.

Among its activities, CETESB conducts environmental poliovirus surveillance through routine sampling and laboratory analysis of seawater from the pier in São Sebastião seaport at São Paulo State cost. No specific permissions are required for developing these activities at this location. This field study did not involve endangered or protected species.

Sampling (Moore swab), concentration (organic flocculation) and processing (chloroform clarification) of samples were performed as described in Sattar & Westwood [15] and USEPA [16]. Poliovirus was isolated according to the WHO alternative test algorithm [17] and submitted to intratypic differentiation using WHO guidelines for Polio laboratories [18].

Individual isolates had been named according to the following convention: PV (poliovirus) followed by the number denoting the type/isolate number/3-letter country followed by the year of isolation (e.g. PV2/44624/BRA2014). For use in this report, the type 2 VDPV isolate name has been shortened to 44624.

### Primary characterization of isolate 44624

The sequences for all primers used in this study are described in S1 Table. Initial virus characterization (intratypic differentiation) was performed using real-time reverse transcription–polymerase chain reaction (rRT-PCR) nucleic acid amplification, provided by the Centers of Disease Control and Prevention–CDC, according to GPLN guidelines [12]. The VDPV screening assay is targeted to nucleotide substitutions that typically revert to the parental WPV sequence during replication of OPV in the human intestine [19]. Viral RNA was extracted from an aliquot of 140μL of L20B cell culture supernatant, using QIAamp Viral RNA Mini Kit (Qiagen, Hilden, Germany) and stored at -80°C for further use. Two different reactions of One-Step Real-time PCR were successively performed in a ABI 7500 Real Time machine (Applied Biosystems, Carlsbad, CA, USA): a Real-time ITD Test, using six set of primers, namely: Pan-Enterovirus, Pan-Poliovirus, Serotype 1, Serotype 2, Serotype 3, and Sabin Multiplex (1, 2 and 3); and a Real-time VDPV Screening Assay, targeting regions in Sabin 1, 2 and 3 known to be involved in reversion to a neurovirulent phenotype. For all Real-Time PCR reactions, the Ct value cutoff for positive reactions was cycle 30.

Candidate VDPVs identified by rRT-PCR screening were sequenced in the VP1 gene (903 nt) for definitive analysis [17,18]. cDNA was prepared from 10 μL of stock viral RNA using 1μl of Superscript II Reverse Transcriptase (Invitrogen, Carlsbad, CA, USA), primed by S2 7439R_Sal (described in S1 Table), and performed at 42°C for 50 minutes, followed by 15 minutes of enzyme inactivation at 70°C. The RNA in the RNA-DNA dimer was specifically degraded by the action of 1μL of Ribonuclease H (Promega, Madison, WI, USA) at 37°C for 30 minutes. The VP1 coding region was amplified and sequenced using the primers Y7/Q8 [20]. The PCR product was purified using QIAquick Gel Extraction Kit (Qiagen, Hilden, Germany), and cycle sequencing reactions were carried out using BigDye terminator chemistry version 3.0 (Applied Biosystems, Carlsbad, CA, USA) on an ABI 3130XL instrument.

### Cross-reactivity using polyclonal antibodies

In order to determine the antigenic characteristics of isolate 44624, a micro neutralization test was performed according to WHO recommendations [21], using serotype-specific PV1, PV2 and PV3 polyclonal antibodies and RD cells. Briefly, two-fold dilutions of polyclonal serum specific for each serotype (WHO/EPI/POLIO) (from 1:2 to 2048) in Eagle-Earle medium were incubated with 100 TCID50 of each poliovirus serotype at 37°C for 1 h. One hundred microliters of Eagle-Earle medium with 2% Fetal Calf Serum, containing 20000 RD cells, were added to the neutralizing mixture (100 ml). Plates were incubated at 37°C for 7 days, and examined for the appearance of CPE with an inverted microscope. Neutralizing antibody titers were calculated by the Karber formula, and expressed as the highest serum dilution neutralizing 50% of the infected cultures.

### Full-length genome amplification

The complete genome of VDPV2 44624 was sequenced for high-resolution analysis. Two long-distance PCR reactions were performed by using the Expand Long Template PCR System (Roche). The two sets of sense/antisense primer pairs 001F_Hind/Q8 and Y7/S2 7439R_Sal were used to amplify two overlapping fragments of 3.57 kb and 5.28 kb, respectively. The PCR mixture for each reaction consisted of 3 μL of cDNA, 300nM sense primer, 300nM antisense primer, 350μM dNTP’s, 5μL 10x PCR buffer 3 with MgCl2, 0,75μl Expand Long Template enzyme mix and DNase/RNase-free water into a 50μl final volume. The cycling parameters for the 3.57kb fragment were 94°C for 2min, 10 cycles of 94°C for 15s, 50°C for 30s and 68°C for 3:30min, followed by 25 cycles of 94°C for 15s, 58°C for 30s and 68°C for 3min30s, with a final extension at 68°C for 10min. For the 5.28kb fragment, the same reaction parameters were used, except for extension periods of 4 minutes and 30 seconds.

The two fragments were purified using QIAquick Gel Extraction Kit (Qiagen, Valencia, CA, USA). Cycle sequencing reactions were carried out using BigDye terminator chemistry version 3.0 (Applied Biosystems, CA, USA), using the primers described in S1 Table. Sequencing was bi-directional, and every nucleotide position was sequenced at least once from each strand. The 3’-end segment sequences were determined by using the 3’ RACE System for Rapid Amplification of cDNA Ends (Life Technologies, USA). Isolate 44624 was also referred to the Centers for Disease Control and Prevention (Atlanta, USA).

The nucleotide and amino acid sequences of isolate 44624 were aligned with the Sabin 2 reference strain (GenBank accession number AY184220) using ClustalW, and molecular evolutionary analyses were performed using MEGA program v6.0 [22]. After analysis of mutational sites, for visualization of the observed amino acid substitutions, the amino acid positions were placed in the 3-dimensional structure model of poliovirus capsid protomer, based on x-ray crystallographic analysis of type 2 poliovirus strain Lansing (PDB ID: 1EAH) [23] using the software Swiss-PdbViewer [24]. The GenBank accession number of isolate 44624 complete genome sequence is KU372652.

### Time of divergence of the isolate 44624 from the original Sabin 2 strain

The time interval between the date of OPV administration and the data of sampling of the VDPV2 44624 was estimated from the nucleotide divergence between the P1/capsid and VP1 sequences of the virus 44624 and Sabin 2 reference strain (GenBank accession number AY184220). The other parts of the genome (containing potential recombination regions) were not included in this analysis. Maximum-likelihood estimates of synonymous substitutions per synonymous sites (K*s*) and nonsynonymous substitutions per nonsynonymous sites (K*a*) were obtained following a modified version of Goldman-Yang codon model of evolution [25] as implemented in MBEToolbox [26]. Total number of substitutions per site (K*t*) was estimated using the T92 model of nucleotide evolution implemented in MBEToolbox [27].

### Recombination events

The aligned full-length genome sequences of isolate 44624 and members of Human Enterovirus C Species (GenBank accession numbers for reference strains Sabin 1 (AY184219), Sabin 2 (AY184220), Sabin 3 (AY184221), Coxsackie virus 1 (CVA1) (AF499635), CVA11 (AF499636), CVA13 (AF465511), CVA17 (AF499639), CVA19 (AF499641), CVA20 (AF499642), CVA21 (AF465515 and D00538), CVA24 (EF026081), Enterovirus C96 (HQ415759), Enterovirus C99 (KJ857508), Enterovirus 104 (JX982259) and Enterovirus C109 (GQ865517)) were subjected to recombination analysis. The RDP4 algorithm package was used to detect homologous recombination events [28] using the default parameters for the methods GENECOV [29], Bootscan [30], Chimaera [31], MaxChi [32], SiScan [33], 3Seq [34] and RDP [35]. As selection criteria for statistical analysis, a putative recombination event was referred to subsequent analysis only when it was consistently identified by at least three of these seven RDP algorithms [36]. The P-value cutoff was chosen as 0.05 and the best signals for recombination are associated with the lowest P-values, which indicates the approximate likelihood for the occurrence of exchange of sequences between genomes (recombination) rather than the probability of convergent evolution of the sequences.

### Phylogenetic analysis

For analysis of divergence and evolution of Brazilian isolate 44624, a phylogenetic tree was constructed for complete VP1 sequences (903nt), using Mega software package, version 6.0 [22]. The 44624 VP1 sequence was aligned to VP1 sequences of Sabin 2 (GenBank accession number AY184220) and a set of divergent type 2 VDPVs identified between 1998–2015, in China (GenBank accession numbers KJ419273-KJ419277, AY948201, HM107832–HM107835) [37–39], Egypt (GenBank accession numbers AF448782 and AF448783) [40], Madagascar (GenBank accession numbers HF913426-HF913428, AM084223, AM084225) [41, 42], Nigeria (GenBank accession numbers JX274980, JX275085, JX274985, JX275162, JX275380, DQ890388) [43, 44], Israel (GenBank accession numbers AJ288062, AM040035-39, AM056049-50, AM158275-6, AM292219-21) [45], Slovakia (GenBank accession numbers JX913541-JX913553, JX913635-913647) [46] and Estonia (GenBank accession numbers KC784367-KC784371) [47].

The evolutionary history was inferred using the Maximum Likelihood Method based on the Kimura-2 parameters [48], the best-fit substitution model indicated by Mega6 software [22] for this dataset. All positions containing gaps and missing data were eliminated.

### Temperature Sensitivity

Reproductive capacity at different temperatures (RCT marker) of isolate 44624 was evaluated on monolayers of RD cells in 6-well plates by an RCT test, in comparison to the Sabin 2 reference strain. A total of 6.5 x 10^5^ cells were seeded per well, and after attachment, 200μL of virus stocks were inoculated and incubated separately at 36.5°C or 40°C for virus absorption for 1 hour, after which the cells were washed, and 3ml of maintenance medium were added per well, followed by incubation at 36.5°C or 40°C, separately. After 8, 24 and 48 h, the cells were harvested, and the TCID50s were calculated in 96-well plates. More than 2 logarithms reduction of the titers at different temperatures was considered to be temperature sensitive [49, 50]. In order to minimize experimental error, the assay was conducted three times.

### One-step growth curve

One-step growth curve experiments were performed in RD cells at 36.5°C and 40°C, with a multiplicity of infection of 10 as determined by TCID50 infectivity titration of virus stocks on RD cells. The used protocol was previously described in [51]. Briefly, 2 x 10^4^ RD cells were added into each well of fourteen 96-well culture plates. After attachment, isolate 44624 and Sabin 2 reference strains were inoculated into three replica wells of 96-well cell culture plates. The plates were incubated at 37°C for 2 hours, and the cells were washed twice with 300 μL of Eagle-Earle medium to remove unbound virus. Then, 100 μL of Eagle-Earle was added per well and plates were incubated at 36.5°C or 40°C for 0, 2, 4, 6, 8, 10 and 12 hours. The plates were subjected to three consecutive freeze-thaw cycles and the viral titers were determined by the TCID50 assay on RD cells at 36.5°C for each time point. In order to guarantee reproducible results, the titration was repeated three independent times for each tested condition.

## Results

### Preliminary Characterization of the Isolate

The virus 44624 was isolated from seawater collected in São Sebastião Seaport, São Paulo, in January 2014. Diagnostic rRT-PCR proved that it is a type 2 VDPV. Compared to Sabin 2 original strain, the isolate 44624 showed 8.6% nucleotide divergence (78/903 nt) in VP1. Among the 9 amino acid changes found, the substitution Ile143Thr, located inside the VP1 DE-loop, is a common substitution in VDPV2 isolates [1, 45, 52], and characterizes the loss of a known major signature of Sabin 2 attenuation [1]. No recombination event was found in the VP1 region.

### Complete sequence of isolate 44624

The full-length genome of isolate 44624 contains 7439 nucleotides plus the polyA tail, with an open reading frame (ORF) of 2207 amino acids. In a comparative analysis with available GenBank sequences, the closest relative in the capsid region appeared to be Sabin 2 strain, although the genetic and amino acid divergences between strains were noteworthy (Table 1). Although the P1 region/capsid is colinear with that of Sabin 2, regions of the 5´-UTR, P2, P3 and 3´-UTR contain sequences derived from other HEV-C Enterovirus.

**Table 1:** Analysis of nucleotide differences between the genome of isolate 44624 and Sabin 2.

Genome region	Nucleotides			Amino acids			
	Length*	Number of nt. substitutions	% Identity	Length**	Number of aa. substitutions#	% Identity	Relevant mutations
5'-UTR	747	128	82.86	NA	NA	NA	5’-UTR hyper variable region; A481G
P1 region	2637	192	92.72	879	14	98.41	
VP4	207	13	93.71	69	0	100	
VP2	813	46	94.34	271	2	99.26	Thr45Ser; Lys152Arg
VP3	714	52	92.71	238	3	98.74	Ser73Asn; Thr75Ala
VP1	903	81	91.03	301	9	99	Ile143Thr; Thr291Ala
P2 region	1725	153	91.13	575	15	97.39	
2A	447	36	91.94	149	4	97.31	
2B	291	57	80.41	97	4	95.88	
2C	987	60	93.92	329	7	97.87	“*cre* element”
P3 region	2259	175	92.25	753	14	98.14	
3A	261	47	81.99	87	4	95.40	
3B	66	11	83.33	22	1	95.45	
3C	366	45	87.70	122	2	98.36	
3D	1557	72	95.37	519	7	98.66	
3´-UTR	71	2	97.18	NA	NA	NA	
Entire genome	7439	650	91.26	2207	43	98.05	

*The figures are for isolate 44624.

**The ORF length was identical for both strains.

NA, not applies.

The 5'-UTR region of 44624 and Sabin 2 have the same size (747 nt.), but differ greatly from each other in the hyper variable region of the 5’-UTR (approximately the last 100nt. before the initiation codon) due to a large number of nucleotide substitutions, insertions and deletions. Position 481 in the 5’-UTR, another major determinant of the attenuated phenotype of Sabin 2, had reverted from A in the Sabin 2 to G found in WPV strains. This transition is frequently observed in VDPV-associated paralytic poliomyelitis cases and is correlated with neurovirulence and efficiency of genomic translation [1, 53].

**Fig 1:**
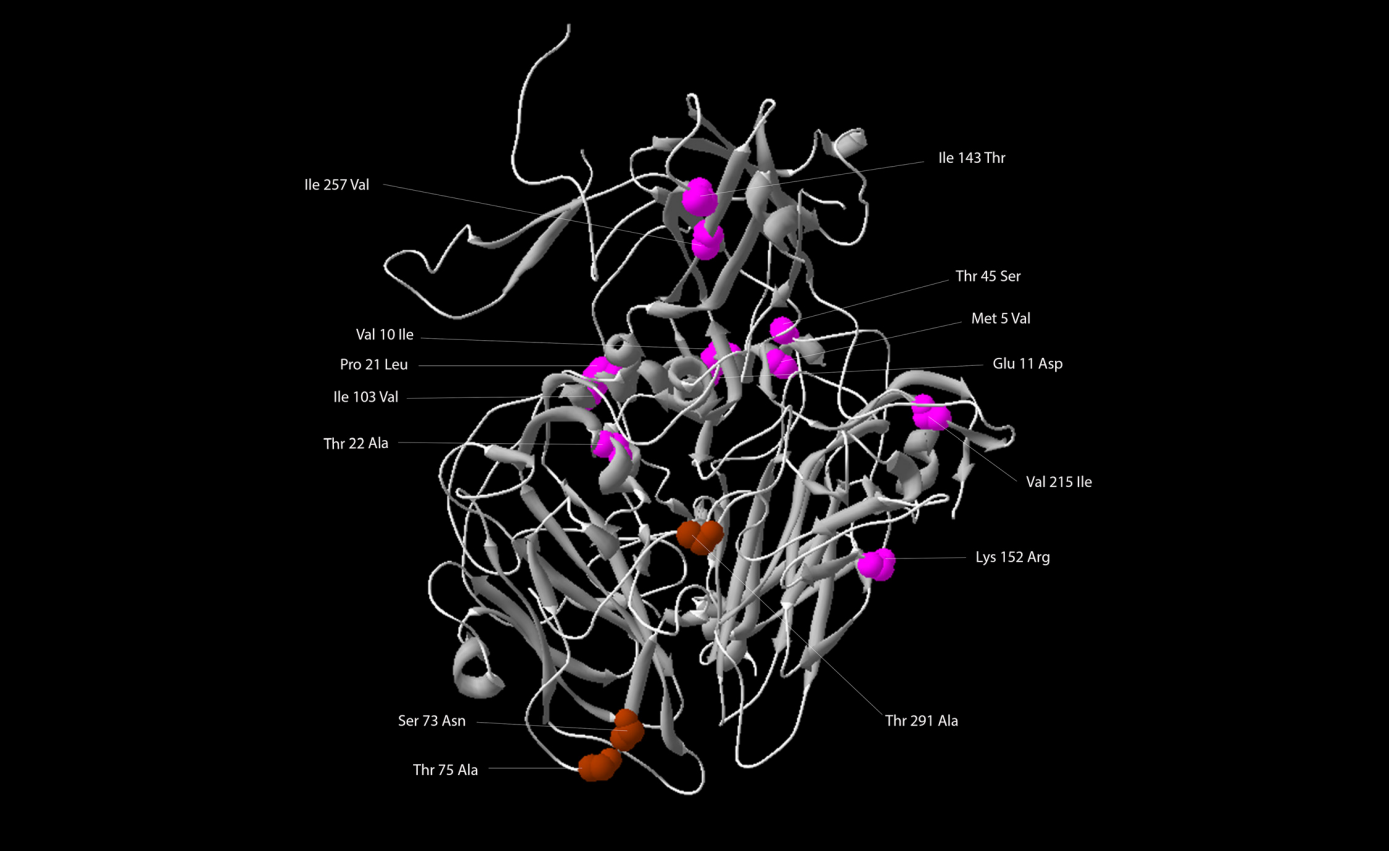
Amino acid substitutions in the capsid protomer of isolate 44624. VP1, VP2, VP3 and VP4 are represented as a 3-dimensional structured protomer. The image was generated using the software Swiss-PdbViewer [24], based on X-ray crystallographic analysis of type 2 poliovirus strain Lansing (Protein Data Bank accession number 1EAH.pdb) [23]. Colour codes: Substitutions at known antigenic sites, brown. Substitutions elsewhere, pink. The BC-loop of VP1 is not visible in this model.

The P1 capsid region contains a total of 188 nt. substitutions, and 14 predicted amino acid substitutions. The mutational pattern of the capsid was characterized by a preponderance of synonymous substitutions (92.02%) over nonsynonymous mutations (7.98%). Nonsynonymous mutations identified in the capsid protomer are show in Fig 1, while the reconstruction of a capsid pentamer (intern and extern view) of isolate 44624 is available in S1 Fig.

Only synonymous mutations were found in VP4. VP2 accounts for 2 nonsynonymous mutations, Thr45Ser and Lys152Arg, the former representing a reversion to the amino acid residues found in both MEF-1 (GenBank accession number AY238473), a laboratory reference wild-type PV2 strain, and Lansing (GenBank accession number M12197), a wild-type PV2 strain associated with fatal paralytic disease in mice after intracerebral inoculation [54–56].

**Fig 2:**
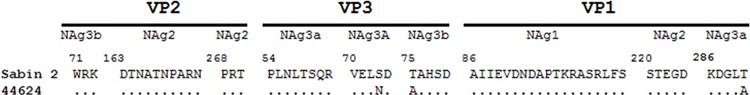
Alignment of amino acids residues of neutralizing antigenic (NAg) sites for Sabin 2 (GenBank accession number AY184220) and isolate 44624. Amino acid positions are numbered according to Sabin 2 NAg1 (VP1 88–106), NAg2 (VP2 163–169; VP2 268–270; VP1 220–225), NAg3a (VP3 54–61; VP3 70–74; VP1 286–291) and NA3b (VP2 71–73; VP3 75–79).

VP3 coding region has three nonsynonymous mutations (Ser73Asn; Thr75Ala; Ile103Val). The substitutions in amino acids 73 and 75 of VP3 are located in NAg 3a and 3b of PV serotype 2, respectively (Fig 2). Another modification in the NAg region was found in VP1 coding region, also in NAg3a (Thr291Ala). VP1 coding region of isolate 44624 contains other 7 nonsynonymous substitutions (Met5Val; Val10Ile; Glu11Asp; Pro9Leu; Thr10Ala; Val215Ile; Ile257Val). In spite of amino acid substitutions inside and near NAg sites, micro neutralization assay with polyclonal PV1-, PV2- and PV3-specific antisera showed that isolate 44624 was completely neutralized by polyclonal PV2-specific antisera, and had no cross-neutralization with PV1- and PV3-specific antisera.

The cis-acting replication element (*cre* element, nt. 4443–4503) in the 2C coding region contained 9 substitutions in comparison with the Sabin 2 reference sequence. Importantly, the Sabin 2 conserved AA**A**CA motif, placed inside the loop of the *cre* structure, was substituted by an AA**G**CA motif in the isolate 44624. The AAGCA motif is found in the type 2 WPV *cre* element [57, 58].

### Analyses of putative recombination events

The complete genome of isolate 44624 and other polio and non-polio HEV-C species were analyzed by using the algorithms available in the RDP package software in search for putative recombination break points. We were able to find five statistically significant (P-value < 0.05) putative recombination events between 44624 genome and other Enterovirus HEV-C reference sequences (Table 2), what strongly indicates that isolate 44624 was a product of recombination between Sabin 2 and other HEV-C viruses.

**Table 2:** Recombination events predicted by RDP algorithms for the genome of isolate 44624 and putative parental sequences. Break points consist of the beginning nucleotide (BN) and the ending nucleotide (EN) of recombination fragment detected in the break point analysis. The figures are for isolate 44624. RDP software considers the major parent as the sequence closely related to that from which the greater part of the recombinant’s sequence may have been derived, and the minor parent is pointed as the sequence closely related to that from which sequences in the proposed recombinant region may have been derived.

Recombinant	Event #	Break points		Parents		Genes	Average P-value						
		BN	EN	Major	Minor		RDP	GENECON	Bootscan	MaxChi	Chimaera	SIScan	3Seq
44624	1	96	509	Sabin 3	CVA24	5’-UTR	1.187x10^-9^	1.059x10^-3^	1.732x10^-11^	2.155x10^-2^	4.668x10^-2^	6.092x10^-7^	4579x10^-9^
	2	744	3797	CVA20	Sabin 2	5’-UTR, VP4, VP2, VP3, VP1, 2A	4.387x10-^55^	2.583x10^-55^	4.658x10^-50^	1.824x10^-20^	2.852x10^-23^	3.291x10^-70^	1.618x10^-10^
	3	744	3771	Sabin 3	Sabin 2	5’-UTR, VP4, VP2, VP3, VP1, 2A	7.153x10^-45^	2.747x10^-42^	9.075x10^-41^	3.372x10^-12^	1.062x10^-21^	5.799x10^-65^	1.715x10^-10^
	4	3305	5578	Unknown—CVA13	CVA20	VP1, 2A, 2B, 2C, 3A, 3B, 3C	2.867x10^-33^	-----	5.708x10^-32^	4.203x10^-12^	1.596x10^-18^	3.572x10^-35^	1.332x10^-14^
	5	3400	5710	CVA13	CVA11	2A, 2B, 2C, 3A, 3B, 3C	1.106x10^-15^	-----	6.333x10^-15^	1.595x10^-9^	1.110x10^-9^	9.698x10^-19^	1.332x10^-14^

The reference sequences of Sabin 2 (AY184220), Sabin 3 (AY184221), CVA11 (AF499636), CVA13 (AF465511), CVA20 (AF499642) and CVA24 (EF026081) were presented as putative parental strains of isolate 44624 by the RDP algorithms (P-value < 0.05). However, it is difficult to precisely determine the parental strains of the isolate 44624 because sometimes two different pairs of major/minor parental strains were predicted with statistical significance for the same genomic region (see predicted recombination events 2 and 3 for VP4-to-VP1 capsid sequences and events 4 and 5 for 2A-to-3C non-structural gene sequences, as described in Table 2).

**Fig 3:**
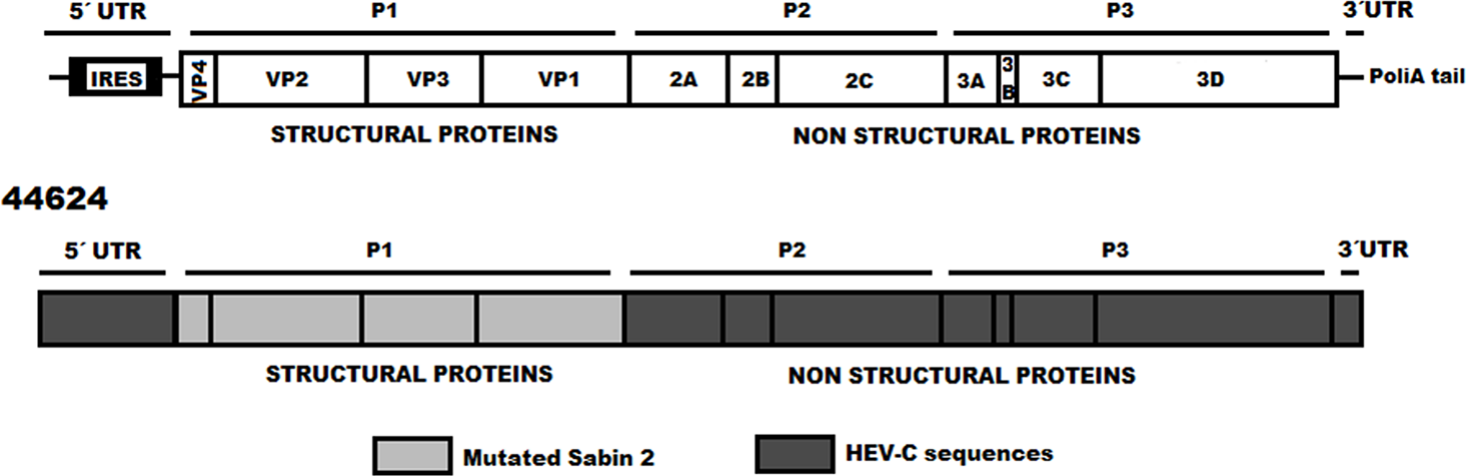
Schematic representation of the genome of recombinant aVDPV 44624, isolated in Brazil. Non-vaccine sequences (HEV-C species) are represented in light blue. Highly mutated Sabin 2 regions are represented in gray.

No close relationship was found by comparing isolate 44624 with sequences belonging to Enterovirus HEV-A, -B or–D species (data not shown). These results suggest that the donors of non-capsid sequences of isolate 44624 were unidentified strains possibly belonging to Enterovirus HEV-C species. A schematic representation of the proposed mosaic genome of 44624 is presented in Fig 3.

### Time of divergence of the isolate 44624 from the Sabin 2 strain

In order to estimate the time of divergence of the isolate 44624 from the original Sabin 2 strain (GenBank accession number AY184220), the approximate evolution time of isolate 44624 relative to Sabin 2 was calculated for both complete P1/capsid and VP1 sequences. It would be an indication of both the date of initial OPV administration and the time of replication of the isolate 44624.

The estimated proportions for *Ks/dS* were 0.249 and 0.301 synonymous substitutions *per* synonymous sites for P1/capsid and VP1 sequences, respectively. The values of 0.008 (P1/capsid) and 0.015 (VP1) were found for nonsynonymous substitutions per nonsynonymous sites (Ka/dN), and the K*t* values were estimated to be 0.077 and 0.094 total substitutions per sites for P1/capsid and VP1 sequences, respectively. The evolution time of isolate 44624 relative to Sabin 2 strain, calculated for the entire P1 region, is about 8.5 years (using K*s* and K*t* clocks), indicating the long time of replication of the isolate 44624 since the OPV initial dose. The calculated value for dN/dS, which can be used as an indicator of selective pressure acting on a protein-coding gene, is 0.033 for P1/capsid (0.051 for VP1 sequence), what is lower than the dN/dS values estimated for iVDPVs datasets (J. Jorba, personal communication).

### Phylogenetic Analysis

**Fig 4:**
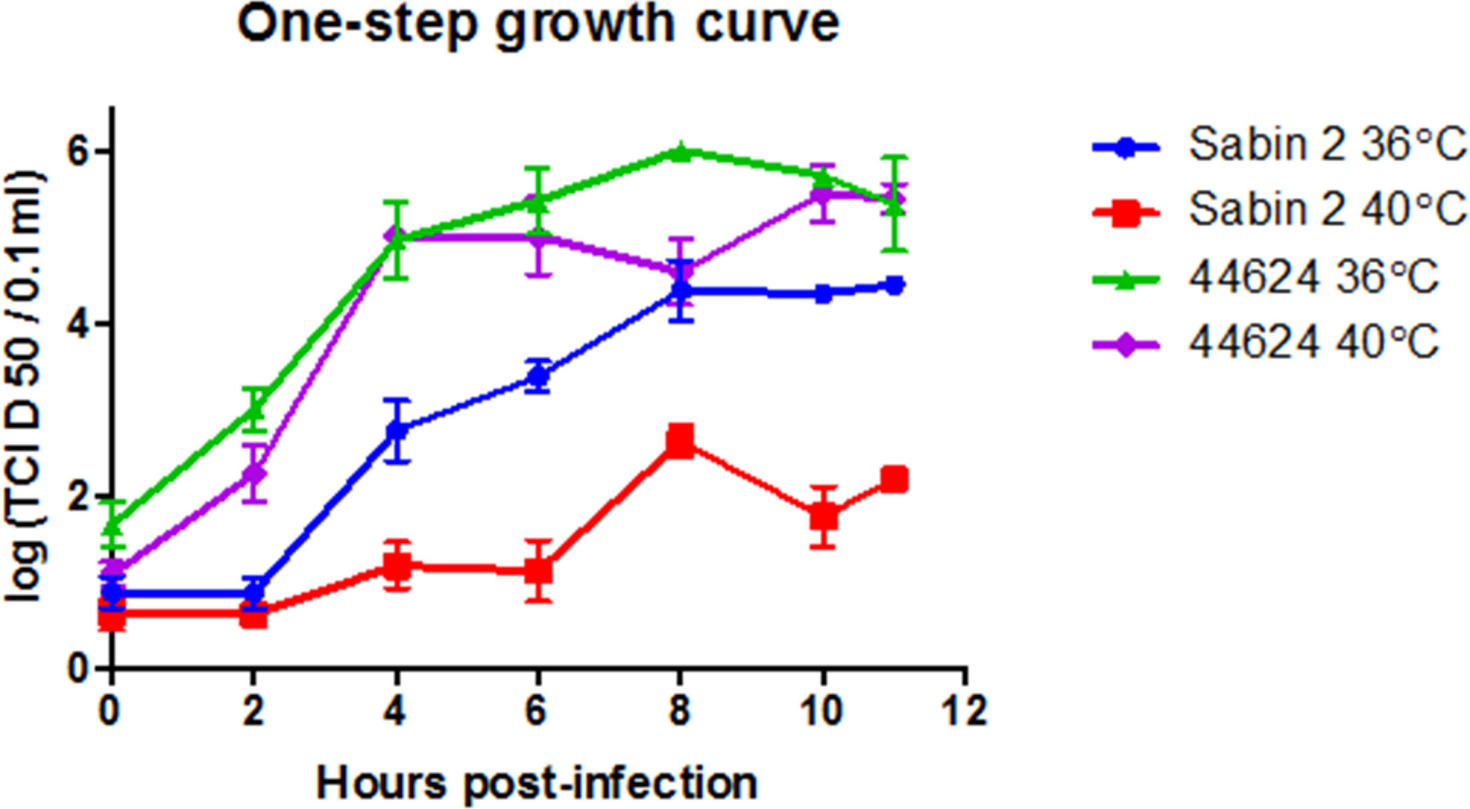
Phylogenetic analysis of VP1 sequences of isolate 44624 and a set of type 2 VDPV isolated between 1998 and 2015. Evolutionary distances were computed using Maximum Composite Likelihood method and Neighbor-joining tree. Consensus from 1000 bootstrap replicates is shown.

The phylogenetic relatedness among isolate 44624 and other VDPV2 sequences available in GenBank was evaluated with MEGA6 software [22]. Phylogenetic investigation revealed no close relationship with the other VDPV analysed (Fig 4); therefore, the geographic origin of the Brazilian VDPV isolate remains unknown.

### RCT marker test

The temperature sensitivity assay showed that there is no significant difference between titer of isolate 44624 at 36.5°C and at 40°C (Table 3), signifying that VDPV2 44624 had lost the attenuated temperature-sensitive (*ts*) phenotype and can efficiently replicate at elevated temperatures, similarly to WPV. For the Sabin 2 reference strain, the >2 log titer difference at 36.5°C and at 40°C is consistent with the *ts* phenotype of Sabin strains. Tests carried out in triplicate showed similar results.

**Table 3:** Reproductive capacity of VDPV2 isolate 44624 and Sabin 2 strain at different temperatures (RCT marker). The RCT value is defined as the difference between the log 10 virus titer of the viral stock measured at the optimal temperature 36,5°C and supraoptimal temperature 40°C. The values are expressed as log 10 TCID50 / 0,1ml. Virus were considered thermosensitive if the ΔRCT value was greater or equal to 2, and thermo resistant when RCT value was inferior to 2.00.

Virus	Hours p.i.	Titer at 36,5°C	Titer at 40°C	Log titer reduction Δ
44624	8h	7.9	7.7	0.2
	24h	7.9	7.9	0.2
	48h	8.1	7.6	0.5
Sabin 2	8h	7.9	3.6	4.3
	24h	7.8	3.8	4.0
	48h	7.7	3.7	4.0

### One-step growth curve

**Fig 5:**
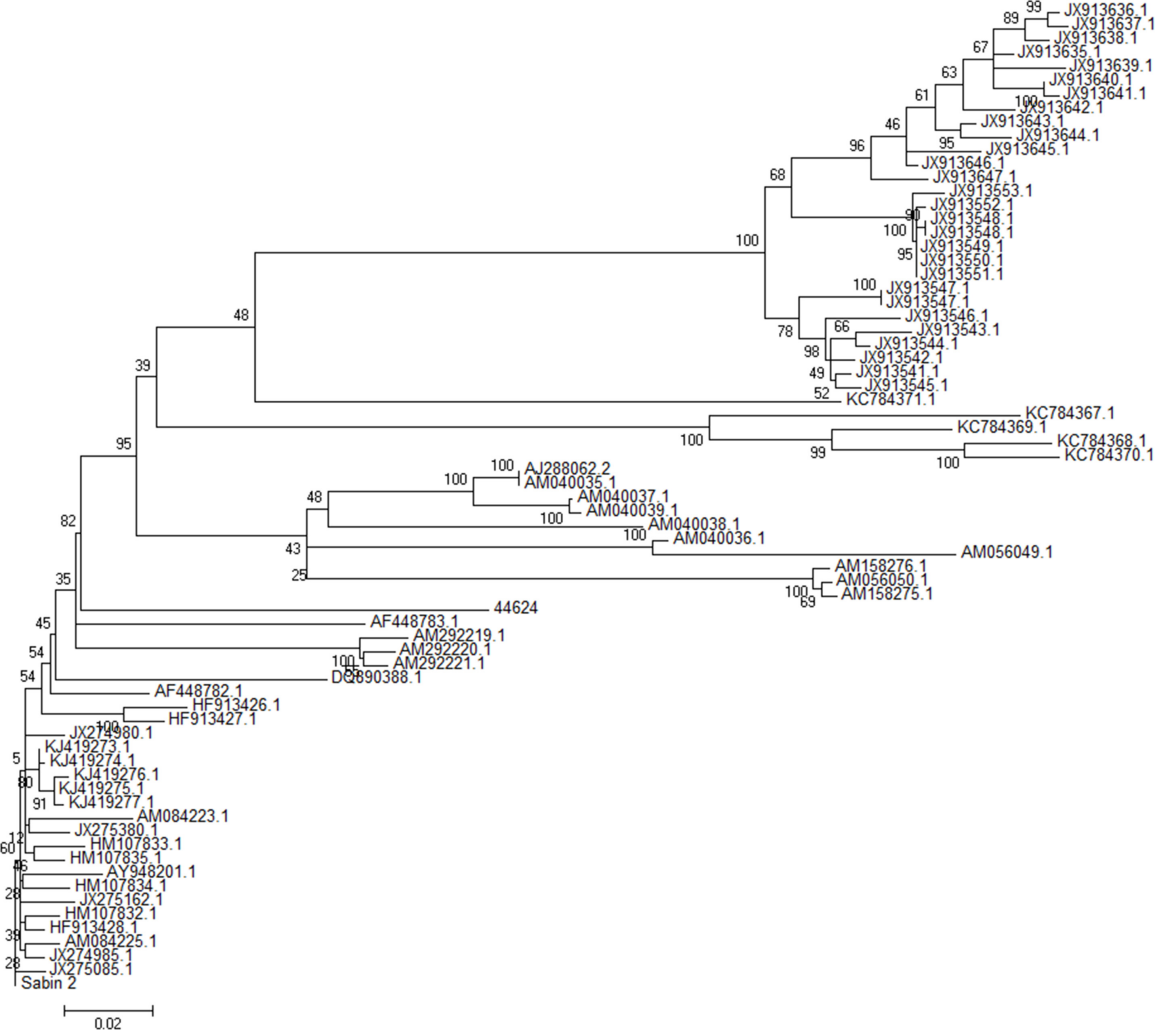
One-step growth curve analysis of isolate 44624 in comparison with Sabin 2 in RD cells. Cells were infected at a MOI of 10 and incubated at 37 or 40°C. Total virus production at different times (0-12h) post-infection were determined by TCID 50 assays on RD cells. Each point represents the mean + standard deviation of virus titers from three different experiments.

The growth rate and virus yields of isolate 44624 were compared with those of Sabin 2 in one-step growth curve experiments at 36.5°C and 40°C in RD cells (Fig 5). The isolate 44624 showed different growth kinetics from that of its Sabin 2 progenitor. At 36.5°C, 44624 had faster growth rates between 4 and 6 h p.i., presenting a curve slope essentially steeper than Sabin 2 strain, what indicates a faster growth rate at the exponential phase of the replication cycle in RD cells. Also, Sabin 2 had a lower final titer at 12 h p.i. At 40°C, 44624 again grew more efficiently between 4 and 6 h. p.i., presenting a result similar of those at 36.5°C, and still a better performance than Sabin 2 at 36,5°C. As expected, Sabin 2 presented lower efficiency of replication at 40°C (*ts* phenotype), and the lowest final titer. As indicated in RCT test, isolate 44624 had lost its temperature sensitivity, and replicated equally well at both tested temperatures. Triplicate tests showed similar results.

## Discussion

This study aimed to assess the molecular and phenotypic characterization of a highly divergent type 2 ambiguous VDPV (aVDPV) isolated from seawater at the São Sebastião Seaport, located on the northern coastline of São Paulo state. The estimates of evolution time of isolate 44624, calculated from the nucleotide divergence from Sabin 2 original strain (AY184220), indicate that the isolate 44624 has been circulating for at least 8.5 years. At the time of 44624 isolation, the overall coverage with 3 doses of oral polio vaccine for children under 1 year old was about ~100% for both Brazil and the town of São Sebastião (Data available at http://pni.datasus.gov.br). The episode was not associated with reported cases of paralytic poliomyelitis.

The last report of indigenous transmission of WPV in Brazil dates back to 1989 [14]. To begin the withdrawal of OPV from the vaccination calendar, Brazil changed to a combined vaccination schedule (2 IPV doses followed by 2 OPV doses) in 2012. Since OPV has been administered in the country for decades, the detection of Sabin strains from the environment is a frequent event. However, it was the first time that a VDPV strain has been reported in Brazil.

The isolate 44624 has no close relationship with either other polio or non-polio Enteroviruses isolated in Brazil before or after this event. Because the seaport is subject to a large flow of people from around the world, it is likely to be an imported virus, with no establishment of a continuous transmission chain in the local population. However, the evidence is insufficient to exclude other possibilities (like the existence of a local chronic excretor).

During evolution, cVDPV and iVDPV are under distinct biological selective pressures, which result in differences in key properties [10]. Isolate 44624 likely has a cVDPV origin, due to the presence of recombination breakpoints with non-vaccine HEV-C Enteroviruses, which frequently occurs during poliovirus circulation and suggests the occurrence of person-to-person transmission [59, 60]. Although it is not mandatory for cVDPV emergence, vaccine/non-vaccine recombination appears to facilitate the replacement of attenuating sequences in a single event [61]. Moreover, iVDPV isolates are widely known to have extensive antigenic variability (many mutations in or near NAg sites), which is not common in cVDPV strains [62] and was not observed in isolate 44624.

The A481G change in the 5’-UTR, along with the amino acid substitution Ile143Thr in VP1 are well known to be the two major determinants of the attenuated phenotype of Sabin 2 [63, 64], and both sites had reverted to the wild-type nt. in isolate 44624. These substitutions are frequently found in VDPV [40, 41, 65] and many OPV-like isolates [63, 66–68], which is probably indicative of the intense selective pressure against these attenuating alleles as OPV replicates in the human intestine [66].

The 44624 recombinant genome has kept only the P1 region/capsid from the original Sabin 2 strain, which determines the antigenic properties of the viral serotype, while all other parts of the genome are derived from recombination with other HEV-C species. As presented in Table 2, between the evaluated sequences of HEV-C species, the strains that statistically are more likely to be parental strains for isolate 44624 are Sabin 2 (AY184220), Sabin 3 (AY184221), CVA11 (AF499636), CVA13 (AF465511), CVA20 (AF499642) and CVA24 (EF026081), but the parental strains could not be precisely determined because there are more than one statistically acceptable event for the majority of putative recombination events. It is possible that more accurate predictions would be achievable through analysis of sequences currently in circulation, instead of reference strains. The absence of a sequence database from HEV-C species currently circulating in Brazil and the impossibility to test the large quantity of HEV-C sequences available at the GenBank were limiting factors of this analysis.

Likewise, phylogenetic analysis did not identify closely related VDPV sequences for isolate 44624. The impracticality to screen all Sabin 2-related sequences available in GenBank and the huge amount of non-deposited sequences were also limitation steps of this analysis. Therefore, the origin of isolate 44624 remains unknown.

The findings presented here indicate that the aVDPV isolate 44624 has been subjected to a long period of circulation and probably person-to-person transmission [10]. The presence of highly drifted, neurovirulent strains in the environment is a potential risk for transmission and spread of pathogenic polioviruses. The findings of this study reiterate the environmental surveillance as a sensitive tool for detection of poliovirus at low levels of circulation and in the absence of paralytic poliomyelitis cases [69–73]. Therefore, the authors encourage the enhancement and expansion of environmental surveillance to help identify any residual transmission in endemic areas and to provide early indication of new importations or emergence of VDPV strains, in order to deliver a world free of polio in the years to come.

## Supporting Information

S1 Fig Location of substituted amino acid sites in 3-dimensional structure model of poliovirus capsid protein pentamer. Visualization is based on x-ray crystallographic analysis of type 2 poliovirus strain Lansing (PDB ID: 1EAH). Panel A, view from outside of virion; Panel B, view from inside the capsid wall. Locations of amino acids substituted in isolate 44624 in comparison to Sabin 2 are indicated. Colour codes: VP1, white; VP2, blue; VP3, cyan; VP4, red. Substitutions at know antigenic sites, brown. Substitutions elsewhere, magenta. The BC-loop of VP1 is not visible in this model.
(PDF)

S1 Table Primers used for complete genomic sequencing of isolate 44624. (DOCX)
