# Supplementary figures and images for: Molecular and Phenotypic Characterization of a Highly Evolved Type 2 Vaccine-Derived Poliovirus Isolated from Seawater in Brazil, 2014
Source: PLoS One. 2016 Mar 28;11(3):e0152251. doi: 10.1371/journal.pone.0152251 (PMC4809597; doi:10.1371/journal.pone.0152251)

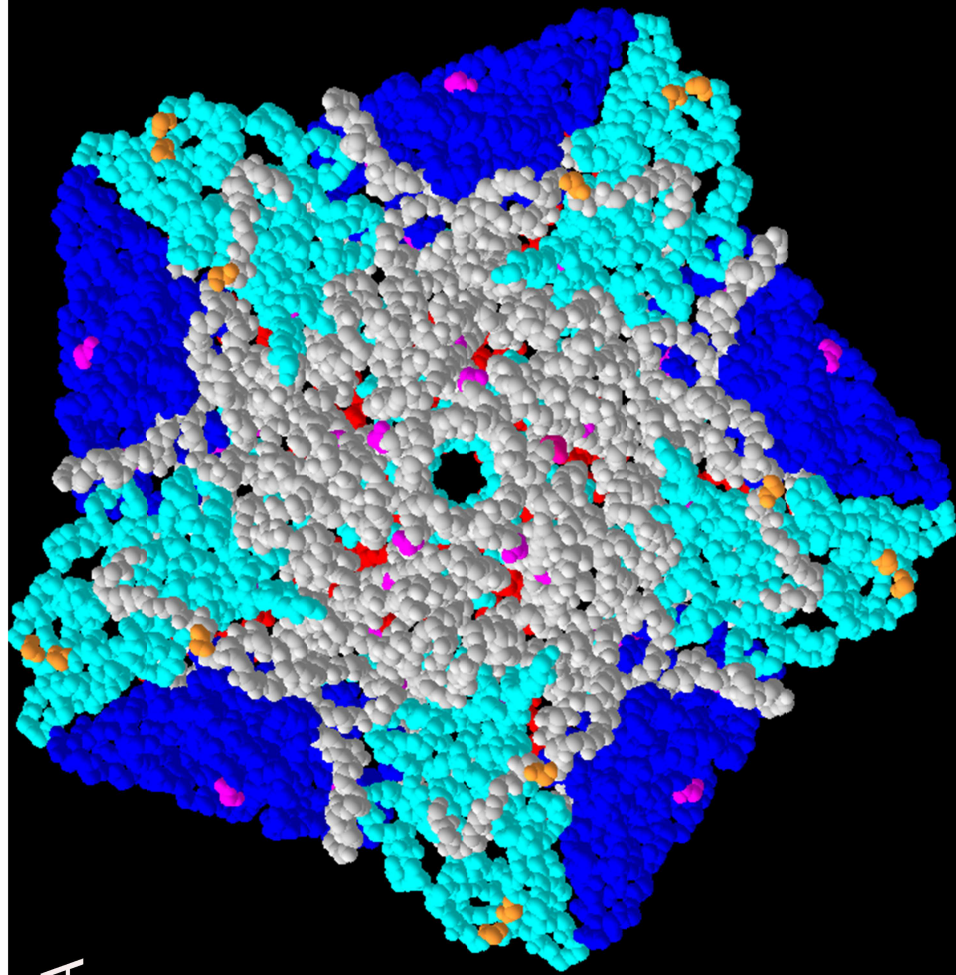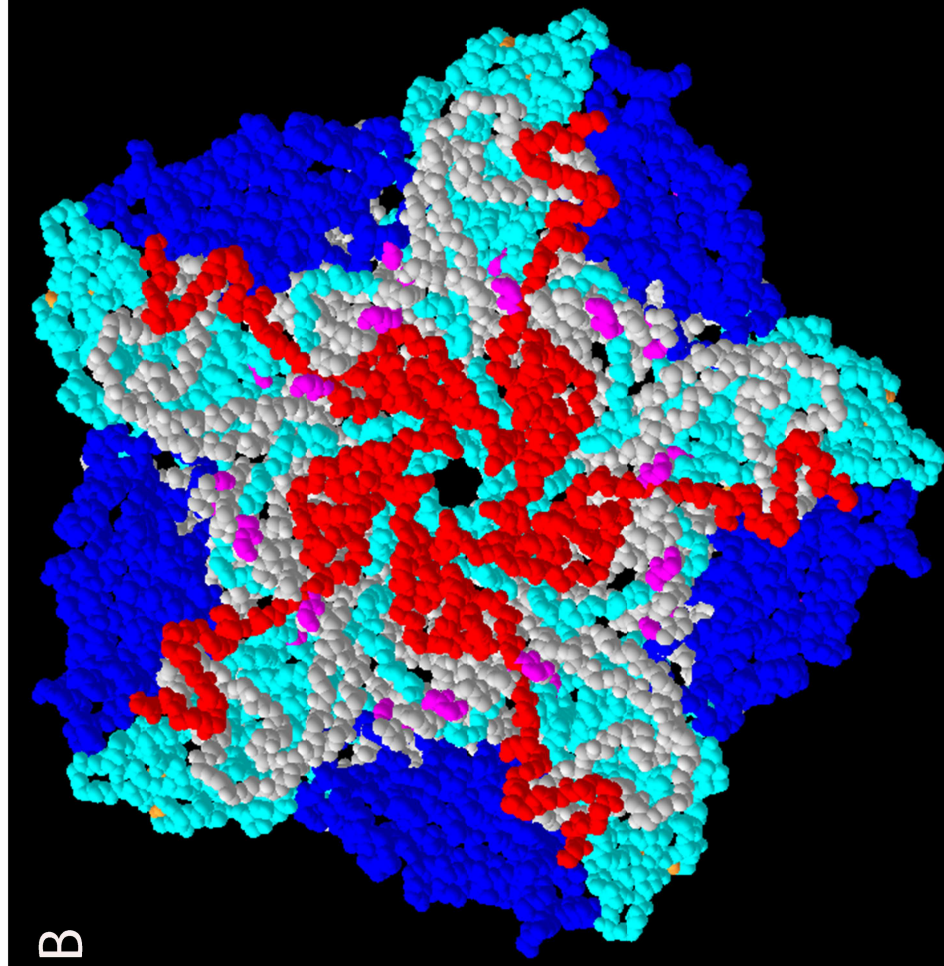

Supplement: S1 Fig — Visualization is based on x-ray crystallographic analysis of type 2 poliovirus strain Lansing (PDB ID: 1EAH). Panel A, view from outside of virion; Panel B, view from inside the capsid wall. Locations of amino acids substituted in isolate 44624 in comparison to Sabin 2 are indicated. Colour codes: VP1, white; VP2, blue; VP3, cyan; VP4, red. Substitutions at know antigenic sites, brown. Substitutions elsewhere, magenta. The BC-loop of VP1 is not visible in this model. (PDF) [file pone.0152251.s001.pdf]
