# Supplementary material for: Molecular and Phenotypic Characterization of a Highly Evolved Type 2 Vaccine-Derived Poliovirus Isolated from Seawater in Brazil, 2014
Source: PLoS One. 2016 Mar 28;11(3):e0152251. doi: 10.1371/journal.pone.0152251 (PMC4809597; doi:10.1371/journal.pone.0152251)
Supplement: S1 Table — (DOCX) [file pone.0152251.s002.docx]

**S1 Table.**  **Primers used for complete genomic sequencing of isolate 44624.**

|  | | |
| --- | --- | --- |
| **Primer** | **Sequence** | **Genome orientation** |
| 001F_Hind | 5’-TCGATGTCGACTAATACGACTCACTATAGGTTAAAACAGCTCTGGGG-3’ | Forward |
| S2 F2 | 5’-CTCCGGCCCCTGAATGCGGCTA-3’ | Forward |
| S2 R1 | 5’-ATTGTCACCATAAGCAGCC-3’ | Reverse |
| S2 F3 | 5’-AGCCTATGGCGGGTCCACCATCAA-3’ | Forward |
| S2 R2 | 5’- TGGTGATCGTTGAATTGCCCAG-3’ | Reverse |
| S2 F4 | 5’-ACAGTGCACGTACAGTGCAATGCTTTCAAG-3’ | Forward |
| S2 R3 | 5’-TTTTTCGCCTGGATTCGCATTCTCGTACTT-3’ | Reverse |
| S2F5 | 5’-TGAACACTCCAGGGAGTAACCA-3’ | Forward |
| S2 R4 | 5’-GGTATCGCACACGGAGACTGGT-3’ | Reverse |
| S2 F6 | 5’-GATAGTTTCACAGAAGGTGGCT-3’ | Forward |
| Y7 | 5′-GGGTTTGTGTCAGCCTGTAATGA-3 | Forward |
| S2 R5 | 5’-CATTGCAAGCTGACACAAA-3’ | Reverse |
| S2 F7 | 5’- CTGTTCAACTGAGACACGCAAACTGGAATTT-3’ | Forward |
| S2 R6 | 5’-GGACGTCTGCCACGTATAGTCATTCCATTT-3’ | Reverse |
| S2 F8 | 5’-GGCTACAAAATTTGCAATTACCAC-3’ | Forward |
| Q8 (S2 R7) | 5′-AAGAGGTCTCTRTTCCACAT-3′ | Reverse |
| S2 F9 | 5’-TCATCACTTGTGATCATTACTA-3’ | Forward |
| S2 R8 | 5’-GCAAGCACTGTGGTAGTGTCCT-3’ | Reverse |
| S2 F10 | 5’-ATTGCTAGAGCAATAGCCGAGA-3’ | Forward |
| S2 R9 | 5’-GGAGGCAGTGAGTATGTGGAGC-3’ | Reverse |
| S2 F11 | 5’-TGATCAGATCACTACAATGATTGTTAATGA-3’ | Forward |
| S2 F12 | 5’-AGATCAGGCAGGAACTAATCTT-3’ | Forward |
| S2 R11 | 5’-GCCTGATATCTCTGAACTTCTC-3’ | Reverse |
| S2 F13 | 5’-CTCAGGACAGACTTTGAAGAAGCAATAT-3’ | Forward |
| S2 R12 | 5’-ATGTCTCTCTTTTTCTTTCCCATTGCTAC-3’ | Reverse |
| S2 F14 | 5’-CCCACCACCTGTACAAAAACAA-3’ | Forward |
| S2 R13 | 5’-TAATCATTGAGTTAAAAATTGA-3’ | Reverse |
| S2 7439R_Sal | 5’-TCGATAAGCTTTTTTTTTTTTTTTTTTTTTTTTTTTCCCCGAATT-3’ | Reverse |

The primers are cited in order of position in the S2 genome.
